# Combinations of Respiratory Chain Inhibitors Have Enhanced Bactericidal Activity against Mycobacterium tuberculosis

**DOI:** 10.1128/AAC.01677-17

**Published:** 2017-12-21

**Authors:** Bryan J. Berube, Tanya Parish

**Affiliations:** a TB Discovery Research, Infectious Disease Research Institute, Seattle, Washington, USA

**Keywords:** antibacterial, bactericidal, respiration, Mycobacterium, tuberculosis

## Abstract

As an obligate aerobe, Mycobacterium tuberculosis uses its electron transport chain (ETC) to produce energy via oxidative phosphorylation. This pathway has recently garnered a lot of attention and is a target for several new antimycobacterials. We tested the respiratory adaptation of M. tuberculosis to phenoxyalkylbenzimidazoles (PABs), compounds proposed to target QcrB, a component of the cytochrome *bc*_1_ complex. We show that M. tuberculosis is able to reroute its ETC to provide temporary resistance to PABs. However, combination treatment of PAB with agents targeting other components of the electron transport chain overcomes this respiratory flexibility. PAB in combination with clofazimine resulted in synergistic killing of M. tuberculosis under both replicating and nonreplicating conditions. PABs in combination with bedaquiline demonstrated antagonism at early time points, particularly under nonreplicating conditions. However, this antagonistic effect disappeared within 3 weeks, when PAB-BDQ combinations became highly bactericidal; in some cases, they were better than either drug alone. This study highlights the potential for combination treatment targeting the ETC and supports the development of PABs as part of a novel drug regimen against M. tuberculosis.

## INTRODUCTION

Mycobacterium tuberculosis is the causative agent of tuberculosis (TB) and the leading cause of death by infectious disease in the world (1). With 1.8 million attributable deaths and nearly 11 million new infections in 2015, TB is not only a major health burden, but it also contributes a societal and economic burden of ∼$12 billion annually (1). In addition, there is an alarming increase in the rate of multidrug-resistant (MDR) and extensively drug-resistant (XDR) M. tuberculosis, which not only lengthens the normal drug regimen from 6 months to 2 years but still causes a mortality rate in treated individuals of ∼50%. Given these facts, there is a dire need for the discovery of novel preventatives and therapeutics to combat M. tuberculosis infections.

M. tuberculosis is an obligate aerobe and, as such, performs respiration for energy production via the electron transport chain (ETC) and oxidative phosphorylation (OxPhos). During OxPhos, M. tuberculosis uses energy derived from nutrients to shuttle electrons through a menaquinone intermediate to oxygen via a branched respiratory chain (2, 3). During replicative growth, electrons are shuttled to a cytochrome *bc*_1_-*aa3*-type oxidase (cyt-*bc*), which is energetically efficient due to its proton-pumping capabilities. As electrons flow through cyt-*bc*, protons are pumped across the membrane to help establish an electrochemical gradient that is used by ATP synthase to generate ATP. Under hypoxic and stress-inducing conditions, electrons can transfer directly to the less energetically favorable but higher-affinity cytochrome *bd* oxidase (cyt-*bd*). Electron transfer to cyt-*bd* does not allow for the efficient establishment of a proton gradient, but it does serve to balance redox equivalents and maintain the proton motive force across the membrane (3).

Recently, much attention has been focused on a group of inhibitors that target different components of the mycobacterial ETC. Bedaquiline, recently approved by the FDA for limited use in MDR-TB patients, targets ATP synthase and presumably inhibits the growth of M. tuberculosis by depleting ATP stores within the cell (4–6). Clofazimine (CFZ), originally an antileprosy drug, serves as a direct competitor of menaquinone by shuttling electrons from the NADH dehydrogenase (NDH-2) to oxygen (7). Upon reoxidation by O_2_, CFZ releases reactive oxygen species (ROS) that kill M. tuberculosis (8). In addition, a number of compound series appear to target QcrB, a component of the cytochrome *bc*_1_ complex, and result in the depletion of intracellular ATP stores (9–11). Of these, the most advanced is Q203, which has recently entered into clinical trials (12).

The phenoxyalkylbenzimidazoles (PABs) are a series of compounds in the early stages of development that have shown promising activity against M. tuberculosis (13). Originally identified from a high-throughput screen (14), PABs are highly adaptable compounds with MICs against M. tuberculosis in the low nanomolar range. PABs exhibit good selectivity for M. tuberculosis, as they inhibit the growth of M. tuberculosis inside macrophages with little to no cytotoxicity against eukaryotic cells (13). We recently identified the probable target of PABs to be QcrB (32), indicating that this series of compounds likely works by inhibiting the cytochrome *bc*_1_ reductase.

Combinations of ETC-targeting compounds have the potential for synergistic activity. For example, combinations of CFZ with BDQ or Q203 demonstrated enhanced killing of M. tuberculosis compared to individual drug treatments (15), thus highlighting the potential efficacy of this approach in establishing new drug regimens. In this study, we tested PABs against a range of strains and confirmed that respiratory flexibility can affect sensitivity to this series, as a strain lacking the cytochrome *bd* oxidase is more sensitive to PABs. However, this respiratory flexibility can be overcome by using PABs in combination with other agents. Here, we demonstrate the synergistic killing of M. tuberculosis with PAB and CFZ against both replicating and nonreplicating bacteria.

## RESULTS

**FIG 1:**
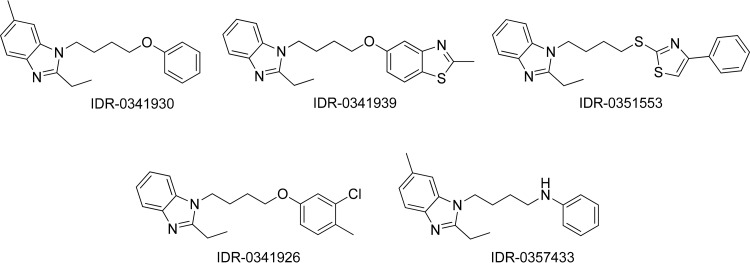
Phenoxyalkylbenzamidazole compounds used in this study.

M. tuberculosis has a respiratory flexibility that responds to the inhibition of QcrB by upregulating the alternative cytochrome *bd* oxidase (15, 16). This response has been seen with several different compound series which apparently target QcrB directly (16). To determine whether this holds true for PAB compounds, we looked at the ability of the PAB series to inhibit bacterial growth against different variants of M. tuberculosis H37Rv (Fig. 1). The key laboratory strains of H37Rv exist as two different ATCC types (ATCC 25618 and ATCC 27294), which have a number of genotypic differences (17). Since previous work demonstrating respiratory remodeling used H37Rv ATCC 27294, we compared the effectiveness of the PAB series against this strain in comparison to the strain in use in our laboratory (ATCC 2618).

Strain H37Rv-MA was slightly more resistant to PAB compounds than strain H37Rv-LP (up to 5-fold increase), but large shifts (>10-fold) were not seen (Table 1). There was also a change in sensitivity to CFZ, BDQ, and rifampin, suggesting that these small changes between parental strains are not specific and may relate to differences in the cell wall. However, in the *cydA*::*aph* mutant strain (in the H37Rv-MA background), PAB compounds were significantly more active than against the parental strain, with a 3- to 9-fold increase in potency. In addition, this strain was also slightly more susceptible to CFZ and BDQ, which target other components of the ETC (Table 1). There was no change in rifampin susceptibility, indicating that the differences in the *cydC*::*aph* mutant strain are not due to a general increase in susceptibility to antimicrobials; rather, this strain is more sensitive to perturbation of the ETC.

**TABLE 1:** Activities of PAB compounds against M. tuberculosis strains

Compound	MIC (μM)^*a*^		
	H37Rv-LP	H37Rv-MA	H37Rv-MA *cydC*::*aph*
IDR-0341930	0.34	0.78	0.14
IDR-0351553	1.1	5.3	0.62
IDR-0341939	1.7	2.7	0.82
IDR-0341926	0.44	1.6	0.18
IDR-0357433	0.34	0.79	0.17
Clofazimine	0.21	0.46	0.19
Bedaquiline	0.24	0.70	0.43
Rifampin	0.008	0.036	0.031

a ETC-targeting compounds and rifampin were tested against select M. tuberculosis strains. MIC is calculated as the concentration at which growth is inhibited by 90%. Data are representative of three independent experiments.

**FIG 2:**
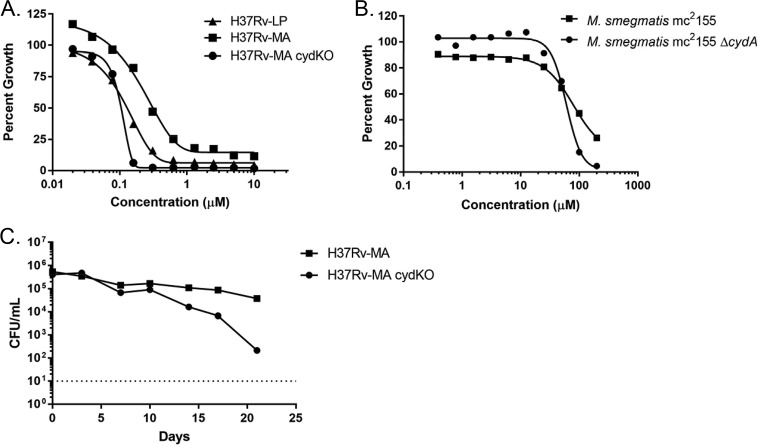
cyt-*bd* confers resistance to PAB compounds. Concentration-response curves for IDR-0341930 against M. tuberculosis (A) or M. smegmatis (B) strains. (C) Kill kinetics of IDR-0341930 against aerobically growing M. tuberculosis H37Rv-MA or H37Rv-MA *cydC*::*aph*. IDR-0341930 was added at 10× the MIC of the individual strains. Data are representative are at least three independent experiments. The dotted line represents the limit of detection (10 bacteria/ml).

Disruption of the *cyd* locus also resulted in a striking change in the shape of the concentration-response curves (CRCs) with PAB compounds. In both parental strains, H37Rv-LP and H37Rv-MA, there were gradual reductions in growth with increasing concentrations of compounds, resulting in a shallow curve, with Hill slopes of −3.13 and −2.57, respectively (Fig. 2A). In contrast, in the *cydC*::*aph* mutant strain, the curves were steeper and achieved complete inhibition of growth at much lower concentrations, with a Hill slope of −8.5 (Fig. 2A). Of interest, the same phenomenon was seen in Mycobacterium smegmatis, a bacterial species generally insensitive to PAB compounds (13). For the wild-type strain, mc^2^155, an MIC could not be calculated, as there was still ∼30% growth at the highest concentration tested (Hill slope, −3.3; Fig. 2B). The M. smegmatis Δ*cydA* mutant strain was more sensitive to IDR-0341930 (MIC, 110 μM) and displayed a steeper slope (Hill slope, −5.4; Fig. 2B). Together, these data indicate that the *cyd* system affects the activities of PAB compounds. The shallowness of the inhibition curves in relation to the *cyd* mutants suggests an adaptive response that allows reduced growth in direct proportion to compound concentration, consistent with the hypothesis that switching to the only other terminal oxidase (cyt-*bd*) occurs and is sufficient to sustain some short-term growth.

We wanted to determine if respiratory remodeling affected compound-mediated bactericidal activity, so we determined the kill kinetics by exposing each strain to 10× their respective MICs. The PAB series is bacteriostatic against aerobically grown replicating M. tuberculosis (13). We confirmed that a representative PAB compound (IDR-0341930) has the same profile against H37Rv-MA, where it was unable to effect a 3-log reduction in 21 days, even at 10× its MIC (Fig. 2C). In the absence of inhibitory compounds, the *cydC*::*aph* mutant strain has growth kinetics identical to the parental strain (data not shown). However, the *cydC*::*aph* mutant was more sensitive to IDR-0341930, with a low rate of kill, resulting in a >3-log reduction in CFU after 21 days (Fig. 2C). These data confirmed the importance of the cytochrome *bd* oxidase as an adaptive response to QcrB inhibition. The dose-response curves in Fig. 2A show that PAB compounds begin to inhibit the growth of H37Rv-MA and the *cydC*::*aph* mutant strain at about the same concentrations. However, there appears to be a concentration of PAB compound above which the *cydC*::*aph* mutant strain is no longer able to grow and instead becomes susceptible to killing by PAB compounds. This strongly suggests that M. tuberculosis uses the cytochrome *bd* oxidase for survival in response to QcrB inhibition.

**FIG 3:**
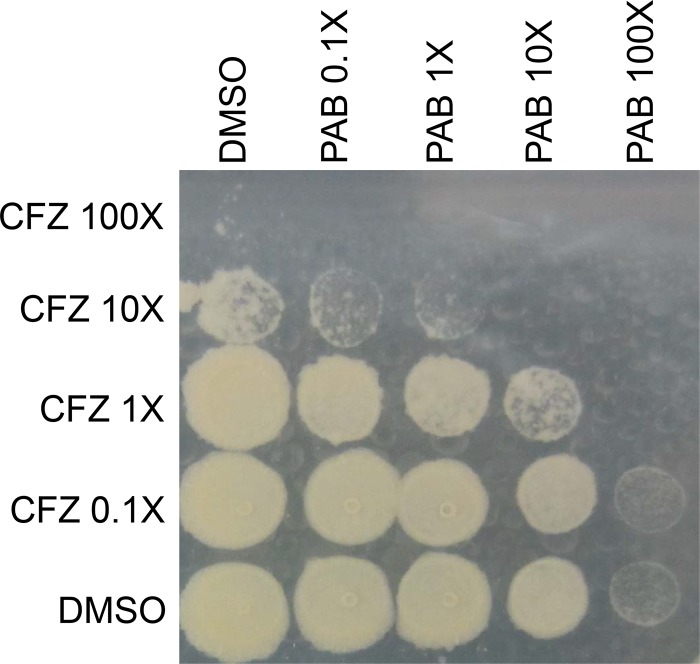
PAB has synergistic bactericidal activity in combination with CFZ. IDR-0341930 and CFZ were tested singly and in combination at the indicated multiples of the MIC against H37Rv-LP for 7 days. Liquid cultures from a 96-well plate were spot-plated onto 7H10-OADC agar. Pictures are representative of two independent experiments.

Since the respiratory adaptation of M. tuberculosis affects the potency of PAB compounds, we hypothesized that we would see synergy with other agents that target the ETC, as these might overcome ETC rerouting. Therefore, we tested whether PABs work synergistically with CFZ and BDQ. We exposed M. tuberculosis H37Rv-LP to drug combinations for 7 days in a 96-well plate and plated it for viability. The PAB compound enhanced the antibacterial activity of CFZ in a range of combinations from 0.1× to 100× the MIC (Fig. 3). IDR-0341930 at 10× the MIC led to reduced viability in combination with either 1× or 10× the MIC of CFZ compared to either IDR-0341930 or CFZ alone (Fig. 3). Even combinations at 1× the MIC of each compound resulted in reduced bacterial viability compared to individual compound treatments (Fig. 3).

**FIG 4:**
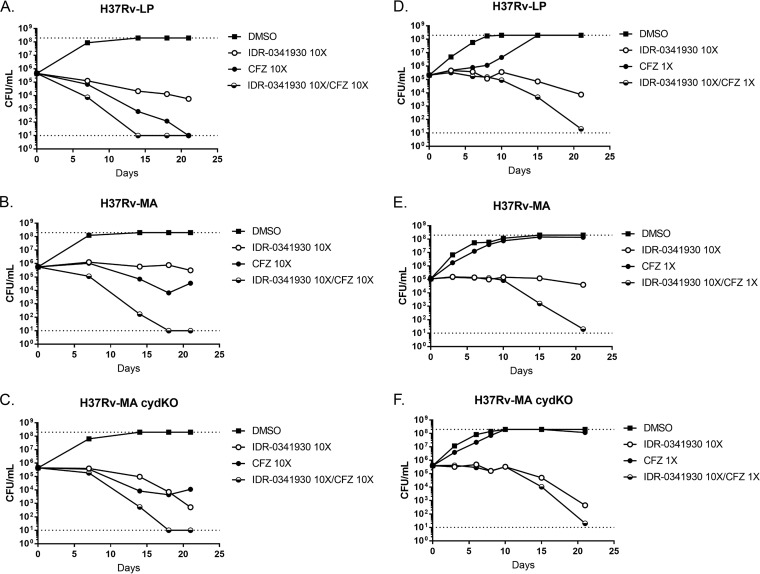
PAB-CFZ combination causes enhanced killing of M. tuberculosis under replicating conditions. Kill kinetics under replicating conditions of IDR-0341930 at 10× the MIC in combination with CFZ at either 10× the MIC (A to C) or 1× the MIC. Combinations were tested against H37Rv-LP (A and D), H37Rv-MA (B and E), or H37Rv-MA *cydC*::*aph* (H37Rv-MA cydKO) (C and F). Samples were taken at the indicated times. Data are representative of at least two independent experiments. The dotted lines represent the upper and lower limits of detection (10^8^ and 10 bacteria/ml, respectively).

To quantify the increased activity of combinations, we performed kill kinetic assays to enumerate viable bacteria after exposure in liquid medium. Compound concentrations were adjusted for each strain depending on their respective MICs. Consistent with our previous data (Fig. 2), IDR-0341930 was bacteriostatic against H37Rv-LP and H37Rv-MA (Fig. 4A and B) but had increased bactericidal activity against H37Rv-MA *cydC*::*aph* (Fig. 4C). In contrast, CFZ at 10× the MIC resulted in complete culture sterilization within 21 days for H37Rv-LP (Fig. 4A). For the H37Rv-MA strains (wild type and *cydC*::*aph* mutant), CFZ did not lead to complete sterilization, with an initial decrease in CFU followed by recovery, possibly due to outgrowth of resistant mutants, upregulation of intrinsic resistance mechanisms, or compound instability (Fig. 2B and C). When given in combination, PAB and CFZ at 10× the MIC were more effective against all three strains, with sterilization of the cultures occurring within 17 days (Fig. 4A to C). At lower concentrations of CFZ roughly equivalent to the MIC, combination with 10× the MIC of PAB led to an increase in bactericidal activity, with almost complete sterilization by 21 days for all three strains. CFZ alone resulted in delayed growth over 10 days but had complete outgrowth by 21 days (Fig. 4D to F). Together, these data highlight the synergistic bactericidal activity of PAB and CFZ in combination.

**FIG 5:**
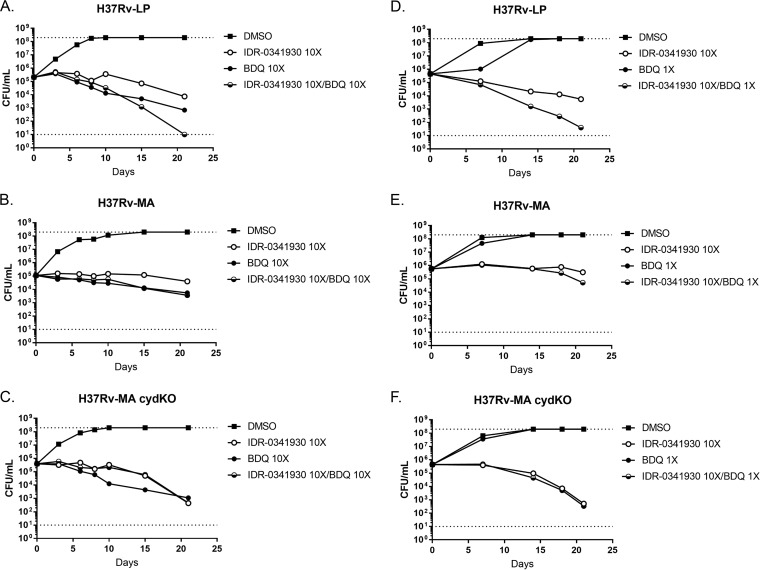
PAB and BDQ show minimal synergistic activity. Kill kinetics under replicating conditions of IDR-0341930 at 10× the MIC in combination with BDQ at either 10× the MIC (A to C) or 1× the MIC. Combinations were tested against H37Rv-LP (A and D), H37Rv-MA (B and E), or H37Rv-MA *cydC*::*aph* (H37Rv-MA cydKO) (C and F). Samples were taken at the indicated times. Data are representative of at least two independent experiments. The dotted lines represent the upper and lower limits of detection (10^8^ and 10 bacteria/ml, respectively).

We next tested IDR-0341930 in combination with BDQ. The addition of IDR-0341930 to BDQ at high concentrations had no effect against the H37Rv-MA strains and a small additive effect against H37Rv-LP at the last time point (Fig. 5A to C). Similarly, at lower concentrations of BDQ, the addition of IDR-0341930 only increased activity against H37Rv-LP but not against the H37Rv-MA strains (Fig. 5D to F). If anything, for the H37Rv-MA *cydC*::*aph* strain, PAB and BDQ were antagonistic at the earliest time points (10 to 15 days), although this effect disappeared by 21 days (Fig. 5C). When IDR-0341930 was tested in combination with rifampin, as a control compound not targeting the ETC, there was no additive activity between the two compounds (data not shown).

**FIG 6:**
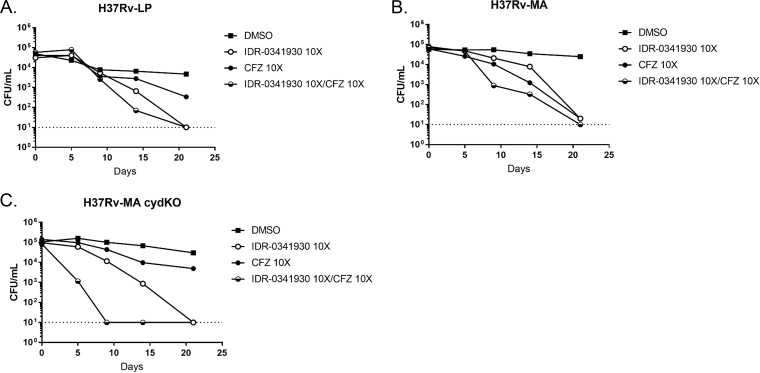
PAB-CFZ combination causes enhanced killing of M. tuberculosis under starvation conditions. Kill kinetics under PBS-starved conditions of IDR-0341930 and CFZ at 10× the MIC against H37Rv-LP (A), H37Rv-MA (B), or H37Rv-MA *cydC*::*aph* (H37Rv-MA cydKO) (C). Data are representative of at least two independent experiments. The dotted line represents the lower limit of detection (10 bacteria/ml).

A major benefit of PAB compounds is their sterilizing activity against nutrient-starved nonreplicating M. tuberculosis (13). We tested whether CFZ or BDQ could enhance this sterilizing activity in M. tuberculosis under starvation. As seen previously, IDR-0341930 had sterilizing activity against H37Rv-LP over a 17-day period (Fig. 6A). CFZ alone was less active, with a <2-log reduction (Fig. 6A). The PAB-CFZ combination treatment had slightly enhanced activity but did not drastically alter the kill kinetics against H37Rv-LP compared to IDR-0341930 alone (Fig. 6A). H37Rv-MA was slightly more resistant to killing by IDR-0341930, as there was an initial delay in kill compared to H37Rv-LP before the strain was sterilized by 21 days (Fig. 6B). Similar to H37Rv-LP, IDR-0341930 had slightly enhanced sterilizing capabilities against H37Rv-MA when given in combination with CFZ (Fig. 6B), although this too was not drastic. When the IDR-0341930–CFZ combination was tested against H37Rv-MA *cydC*::*aph*, there was a drastic reduction in CFU with sterilization of the culture in less than 10 days (Fig. 6C). Taken together, these data show that CFZ and PAB compounds have synergistic activity under both replicating and nonreplicating conditions and that this combination can overcome any respiratory remodeling in the bacteria.

**FIG 7:**
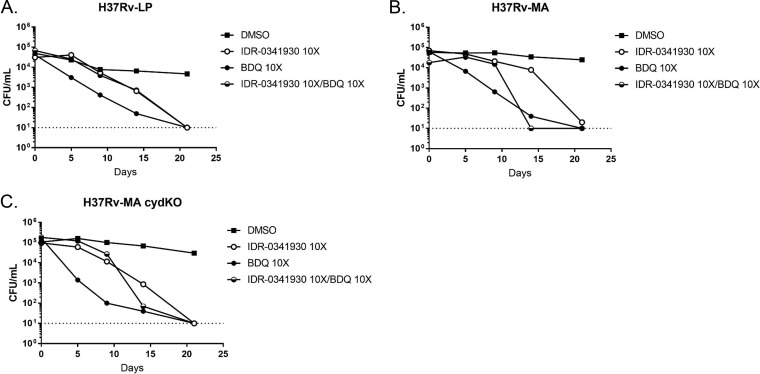
PAB-BDQ combinations are active under starvation conditions but are antagonistic at early time points. Kill kinetics under PBS-starved conditions of IDR-0341930 and BDQ at 10× the MIC against H37Rv-LP (A), H37Rv-MA (B), or H37Rv-MA *cydC*::*aph* (H37Rv-MA cydKO) (C). Data are representative of at least two independent experiments. The dotted line represents the lower limit of detection (10 bacteria/ml).

We also tested BDQ in combination with PAB. As expected from an agent with activity against nonreplicating bacteria (6), we saw a time-dependent decrease in bacterial viability for all three strains treated with BDQ (Fig. 7). Surprisingly, we saw antagonism between BDQ and PAB at early time points, with bacteria surviving better under combination treatment than with individual drugs. For all strains, this antagonism disappeared within 2 to 3 weeks, at which point all cultures became sterilized (Fig. 7).

## DISCUSSION

The mycobacterial electron transport chain has garnered significant attention as a novel pathway for chemical inhibition. M. tuberculosis can adapt to changing environmental conditions or chemical inhibition of the cyt-*bc* by switching the respiratory pathway to use of the cytochrome *bd* oxidase (15, 16, 18). We show that this respiratory flexibility also occurs in response to treatment with PAB compounds, and a strain lacking cytochrome *bd* is more sensitive to both inhibition of growth and killing by PABs.

Any new treatment for M. tuberculosis is sure to be given in combination with other antimycobacterial drugs. This opens the possibility of combination therapies targeting the ETC, which would have the dual benefit of limiting the possibility of resistant mutant generation and counteracting M. tuberculosis respiratory flexibility and tolerance networks (2, 19). Kalia and colleagues recently provided support for this idea by describing a synthetic lethal relationship in a mouse model of infection when using Q203 to target QcrB in a strain with a genetic knockout of cyt-*bd* (20). Additionally, a combination of Q203 and CFZ is more active against M. tuberculosis cultures than either compound alone (15). Our data support the concept that any inhibitor of QcrB would have synergy with CFZ, as we see similar results with an entirely different QcrB-targeting compound series. We also demonstrate that this synergy occurs under nonreplicating conditions as well. This is an important finding due to the heterogeneity among lesion types found in both human and mouse infections where bacteria can reside in distinct microenvironments containing various levels of nutrients and/or oxygen tensions (21, 22). Drug combinations active against multiple states of M. tuberculosis growth and/or persistence will allow for enhanced killing and a reduced rate of resistant mutant generation.

A major benefit of PAB-CFZ combination treatment is that the compounds appear to work synergistically against M. tuberculosis across a range of concentrations, and this effect is seen with more than one method of measurement. The fact that this synergy is seen both at low (including subinhibitory concentrations) and high (10× the inhibitory concentrations) concentrations is important, since during treatment, the effective concentrations of drugs at the infection site are likely to vary. It was interesting to note that at lower concentrations of CFZ, there was a delay in the time before bacterial death was observed. This delay is likely due to initial rerouting of the ETC following chemical inhibition of QcrB and/or NDH-2, which can maintain viability over a short time. However, with sustained treatment, the lethal effect of the drug combination eventually overcomes any respiratory adaptation.

We saw minimal synergy between PAB and BDQ, consistent with previous reports that a Q203-BDQ combination had kill kinetics nearly identical to that of BDQ alone (15). However, we did see antagonism between IDR-0341930 and BDQ at early time points, particularly under starvation conditions (Fig. 5 and 7). This is surprising, since QcrB inhibitors result in reduced intracellular ATP levels (11, 23) and so seem to be ideal candidates for combination treatment with BDQ. However, similar effects have been seen in M. smegmatis, where mutant strains with a deletion of the cytochrome *bc*_1_ system are less sensitive to BDQ (24). Additionally, BDQ treatment leads to a transient increase in ATP levels in M. tuberculosis, possibly due to rerouting of respiration or increases in substrate-level phosphorylation. This effect may account for the antagonism. BDQ may also have other effects, and recent data suggest it can act as an uncoupler, allowing proton flow across the membrane without the benefit of ATP production (25). An alternative possibility is that the upregulation of the *bd* oxidase induced by BDQ could antagonize PAB activity (16, 26, 27); since Q203 also induced *bd* oxidase, the combination might lead to even greater expression than either drug treatment alone, in the process conferring enhanced protection to PAB-BDQ treatment until ATP levels are sufficiently suppressed so as to cause bacterial killing. Understanding the precise mechanisms of action of the different compounds will be important to predict their effect in combination. These different possibilities are under investigation.

The work described herein highlights combination drug treatments that are highly effective at killing M. tuberculosis under both aerobic and starvation conditions, particularly PAB-CFZ. Future work will need to focus on understanding the true extent of respiratory flexibility of the mycobacterial ETC in order to determine the optimal combination of ETC-targeting compounds. More detailed understanding, including transcriptional profiling, of the rerouting of the ETC in response to chemical or genetic perturbations will allow for the rational design of combination treatments to most effectively kill M. tuberculosis.

## MATERIALS AND METHODS

### Bacterial strains and growth conditions.

M. tuberculosis H37Rv-LP (London Pride) (ATCC 25618) (17), H37Rv-MA (ATCC 27294) (provided by Chris Sassetti), H37Rv-MA *cydC*::*aph* (28) (provided by Helena Boshoff), M. smegmatis mc^2^155, and M. smegmatis mc^2^155 Δ*cydA* (29) (provided by Bavesh Kana) were used in this study. Mycobacterial strains were grown in Middlebrook 7H9 medium containing 10% (vol/vol) oleic acid-albumin-dextrose-catalase (OADC) supplement (Becton Dickinson) and 0.05% (wt/vol) Tween 80 (7H9-Tw-OADC) under aerobic conditions.

### Determination of MIC.

MICs were determined as previously described (30); briefly, MICs were determined against M. tuberculosis and M. smegmatis strains grown in 7H9-Tw-OADC under aerobic conditions. M. tuberculosis growth was measured by the optical density at 590 nm (OD_590_) after 5 days of incubation at 37°C, while M. smegmatis growth was measured after 24 h. The MIC is defined as the concentration of compound required to inhibit the growth of M. tuberculosis by 90% and was determined from the Levenberg-Marquardt least-squares plot (31).

### Determination of compound kill kinetics.

Compound kill kinetics were performed as previously described (13). Briefly, M. tuberculosis was grown in 7H9-Tw-OADC. Standing cultures were inoculated with 1 × 10^5^ CFU/ml and compound at the indicated dose (final dimethyl sulfoxide [DMSO] concentration of 2%). Starved cells were generated by resuspending M. tuberculosis in phosphate-buffered saline plus 0.05% tyloxapol (PBS-Ty) at 1 × 10^5^ CFU/ml and incubating at 37°C standing for 2 weeks before adding compounds. Cultures were serially diluted and plated onto 7H10-OADC agar, and the CFU were counted after 3 to 4 weeks of incubation at 37°C. For the spot-based assay, bacteria and compounds were cultured in 96-well plates for 7 days. Cultures were diluted 1:10 using a plate stamper (Sorenson Biosciences), and 5 μl from each well was stamped onto 7H10-OADC agar.

## References

[1] World Health Organization. 2016 Global tuberculosis report 2016. World Health Organization, Geneva, Switzerland http://apps.who.int/iris/bitstream/10665/250441/1/9789241565394-eng.pdf?ua=1.

[2] Bald D, Villellas C, Lu P, Koul A 2017 Targeting energy metabolism in Mycobacterium tuberculosis, a new paradigm in antimycobacterial drug discovery. mBio 8(2):e00272-17. doi:10.1128/mBio.00272-17. 28400527 PMC5388804

[3] Cook GM, Hards K, Dunn E, Heikal A, Nakatani Y, Greening C, Crick DC, Fontes FL, Pethe K, Hasenoehrl E, Berney M 2017 Chapter 14: Oxidative phosphorylation as a target space for tuberculosis: success, caution, and future directions, p 295–316. *In* Jacobs W Jr, McShane H, Mizrahi V, Orme I (ed),Tuberculosis and the tubercle bacillus, 2nd ed ASM Press, Washington, DC. doi:10.1128/microbiolspec.TBTB2-0014-2016. PMC5480969 28597820

[4] Andries K, Verhasselt P, Guillemont J, Gohlmann HW, Neefs JM, Winkler H, Van Gestel J, Timmerman P, Zhu M, Lee E, Williams P, de Chaffoy D, Huitric E, Hoffner S, Cambau E, Truffot-Pernot C, Lounis N, Jarlier V 2005 A diarylquinoline drug active on the ATP synthase of Mycobacterium tuberculosis. Science 307:223–227. doi:10.1126/science.1106753. 15591164

[5] Koul A, Dendouga N, Vergauwen K, Molenberghs B, Vranckx L, Willebrords R, Ristic Z, Lill H, Dorange I, Guillemont J, Bald D, Andries K 2007 Diarylquinolines target subunit c of mycobacterial ATP synthase. Nat Chem Biol 3:323–324. doi:10.1038/nchembio884. 17496888

[6] Koul A, Vranckx L, Dendouga N, Balemans W, Van den Wyngaert I, Vergauwen K, Gohlmann HW, Willebrords R, Poncelet A, Guillemont J, Bald D, Andries K 2008 Diarylquinolines are bactericidal for dormant mycobacteria as a result of disturbed ATP homeostasis. J Biol Chem 283:25273–25280. doi:10.1074/jbc.M803899200. 18625705

[7] Lechartier B, Cole ST 2015 Mode of action of clofazimine and combination therapy with benzothiazinones against Mycobacterium tuberculosis. Antimicrob Agents Chemother 59:4457–4463. doi:10.1128/AAC.00395-15. 25987624 PMC4505229

[8] Yano T, Kassovska-Bratinova S, Teh JS, Winkler J, Sullivan K, Isaacs A, Schechter NM, Rubin H 2011 Reduction of clofazimine by mycobacterial type 2 NADH:quinone oxidoreductase: a pathway for the generation of bactericidal levels of reactive oxygen species. J Biol Chem 286:10276–10287. doi:10.1074/jbc.M110.200501. 21193400 PMC3060482

[9] Abrahams KA, Cox JA, Spivey VL, Loman NJ, Pallen MJ, Constantinidou C, Fernandez R, Alemparte C, Remuinan MJ, Barros D, Ballell L, Besra GS 2012 Identification of novel imidazo[1,2-*a*]pyridine inhibitors targeting *M. tuberculosis* QcrB. PLoS One 7:e52951. doi:10.1371/journal.pone.0052951. 23300833 PMC3534098

[10] Moraski GC, Seeger N, Miller PA, Oliver AG, Boshoff HI, Cho S, Mulugeta S, Anderson JR, Franzblau SG, Miller MJ 2016 Arrival of imidazo[2,1-*b*]thiazole-5-carboxamides: potent anti-tuberculosis agents that target QcrB. ACS Infect Dis 2:393–398. doi:10.1021/acsinfecdis.5b00154. 27627627

[11] Pethe K, Bifani P, Jang J, Kang S, Park S, Ahn S, Jiricek J, Jung J, Jeon HK, Cechetto J, Christophe T, Lee H, Kempf M, Jackson M, Lenaerts AJ, Pham H, Jones V, Seo MJ, Kim YM, Seo M, Seo JJ, Park D, Ko Y, Choi I, Kim R, Kim SY, Lim S, Yim SA, Nam J, Kang H, Kwon H, Oh CT, Cho Y, Jang Y, Kim J, Chua A, Tan BH, Nanjundappa MB, Rao SP, Barnes WS, Wintjens R, Walker JR, Alonso S, Lee S, Kim J, Oh S, Oh T, Nehrbass U, Han SJ, No Z, 2013 Discovery of Q203, a potent clinical candidate for the treatment of tuberculosis. Nat Med 19:1157–1160. doi:10.1038/nm.3262. 23913123

[12] Task Foundation NPC. 2015 Phase 2 trial to evaluate the early bactericidal activity, safety and tolerability of meropenem plus amoxycillin/CA and faropenem plus amoxycillin/CA in adult patients with newly diagnosed pulmonary tuberculosis. https://clinicaltrials.gov/ct2/show/NCT02349841.

[13] Chandrasekera NS, Alling T, Bailey MA, Files M, Early JV, Ollinger J, Ovechkina Y, Masquelin T, Desai PV, Cramer JW, Hipskind PA, Odingo JO, Parish T 2015 Identification of phenoxyalkylbenzimidazoles with antitubercular activity. J Med Chem 58:7273–7285. doi:10.1021/acs.jmedchem.5b00546. 26295286

[14] Ananthan S, Faaleolea ER, Goldman RC, Hobrath JV, Kwong CD, Laughon BE, Maddry JA, Mehta A, Rasmussen L, Reynolds RC, Secrist JA III, Shindo N, Showe DN, Sosa MI, Suling WJ, White EL 2009 High-throughput screening for inhibitors of Mycobacterium tuberculosis H37Rv. Tuberculosis (Edinb) 89:334–353. doi:10.1016/j.tube.2009.05.008. 19758845 PMC3255569

[15] Lamprecht DA, Finin PM, Rahman MA, Cumming BM, Russell SL, Jonnala SR, Adamson JH, Steyn AJ 2016 Turning the respiratory flexibility of Mycobacterium tuberculosis against itself. Nat Commun 7:12393. doi:10.1038/ncomms12393. 27506290 PMC4987515

[16] Arora K, Ochoa-Montano B, Tsang PS, Blundell TL, Dawes SS, Mizrahi V, Bayliss T, Mackenzie CJ, Cleghorn LA, Ray PC, Wyatt PG, Uh E, Lee J, Barry CE III, Boshoff HI 2014 Respiratory flexibility in response to inhibition of cytochrome C oxidase in Mycobacterium tuberculosis. Antimicrob Agents Chemother 58:6962–6965. doi:10.1128/AAC.03486-14. 25155596 PMC4249445

[17] Ioerger TR, Feng Y, Ganesula K, Chen X, Dobos KM, Fortune S, Jacobs WR Jr, Mizrahi V, Parish T, Rubin E, Sassetti C, Sacchettini JC 2010 Variation among genome sequences of H37Rv strains of Mycobacterium tuberculosis from multiple laboratories. J Bacteriol 192:3645–3653. doi:10.1128/JB.00166-10. 20472797 PMC2897344

[18] Moosa A, Lamprecht DA, Arora K, Barry CE III, Boshoff HIM, Ioerger TR, Steyn AJC, Mizrahi V, Warner DF 2017 Susceptibility of *Mycobacterium tuberculosis* cytochrome *bd* oxidase mutants to compounds targeting the terminal respiratory oxidase, cytochrome *c*. Antimicrob Agents Chemother 61:e01338-17. doi:10.1128/AAC.01338-17. 28760899 PMC5610507

[19] Peterson EJR, Ma S, Sherman DR, Baliga NS 2016 Network analysis identifies Rv0324 and Rv0880 as regulators of bedaquiline tolerance in Mycobacterium tuberculosis. Nat Microbiol 1:16078. doi:10.1038/nmicrobiol.2016.78. 27573104 PMC5010021

[20] Kalia NP, Hasenoehrl EJ, Ab Rahman NB, Koh VH, Ang MLT, Sajorda DR, Hards K, Gruber G, Alonso S, Cook GM, Berney M, Pethe K 2017 Exploiting the synthetic lethality between terminal respiratory oxidases to kill Mycobacterium tuberculosis and clear host infection. Proc Natl Acad Sci U S A 114:7426–7431. doi:10.1073/pnas.1706139114. 28652330 PMC5514758

[21] Irwin SM, Prideaux B, Lyon ER, Zimmerman MD, Brooks EJ, Schrupp CA, Chen C, Reichlen MJ, Asay BC, Voskuil MI, Nuermberger EL, Andries K, Lyons MA, Dartois V, Lenaerts AJ 2016 Bedaquiline and pyrazinamide treatment responses are affected by pulmonary lesion heterogeneity in Mycobacterium tuberculosis infected C3HeB/FeJ mice. ACS Infect Dis 2:251–267. doi:10.1021/acsinfecdis.5b00127. 27227164 PMC4874602

[22] Lenaerts A, Barry CE III, Dartois V 2015 Heterogeneity in tuberculosis pathology, microenvironments and therapeutic responses. Immunol Rev 264:288–307. doi:10.1111/imr.12252. 25703567 PMC4368385

[23] Rybniker J, Vocat A, Sala C, Busso P, Pojer F, Benjak A, Cole ST 2015 Lansoprazole is an antituberculous prodrug targeting cytochrome *bc*_1_. Nat Commun 6:7659. doi:10.1038/ncomms8659. 26158909 PMC4510652

[24] Lu P, Heineke MH, Koul A, Andries K, Cook GM, Lill H, van Spanning R, Bald D 2015 The cytochrome *bd*-type quinol oxidase is important for survival of *Mycobacterium smegmatis* under peroxide and antibiotic-induced stress. Sci Rep 5:10333. doi:10.1038/srep10333. 26015371 PMC4450806

[25] Hards K, Robson JR, Berney M, Shaw L, Bald D, Koul A, Andries K, Cook GM 2015 Bactericidal mode of action of bedaquiline. J Antimicrob Chemother 70:2028–2037. doi:10.1093/jac/dkv054. 25754998

[26] Koul A, Vranckx L, Dhar N, Gohlmann HW, Ozdemir E, Neefs JM, Schulz M, Lu P, Mortz E, McKinney JD, Andries K, Bald D 2014 Delayed bactericidal response of Mycobacterium tuberculosis to bedaquiline involves remodelling of bacterial metabolism. Nat Commun 5:3369. doi:10.1038/ncomms4369. 24569628 PMC3948051

[27] Small JL, Park SW, Kana BD, Ioerger TR, Sacchettini JC, Ehrt S 2013 Perturbation of cytochrome *c* maturation reveals adaptability of the respiratory chain in *Mycobacterium tuberculosis*. mBio 4(5):e00475-13. doi:10.1128/mBio.00475-13. 24045640 PMC3781833

[28] Shi L, Sohaskey CD, Kana BD, Dawes S, North RJ, Mizrahi V, Gennaro ML 2005 Changes in energy metabolism of *Mycobacterium tuberculosis* in mouse lung and under *in vitro* conditions affecting aerobic respiration. Proc Natl Acad Sci U S A 102:15629–15634. doi:10.1073/pnas.0507850102. 16227431 PMC1255738

[29] Kana BD, Weinstein EA, Avarbock D, Dawes SS, Rubin H, Mizrahi V 2001 Characterization of the *cydAB*-encoded cytochrome *bd* oxidase from *Mycobacterium smegmatis*. J Bacteriol 183:7076–7086. doi:10.1128/JB.183.24.7076-7086.2001. 11717265 PMC95555

[30] Ollinger J, Bailey MA, Moraski GC, Casey A, Florio S, Alling T, Miller MJ, Parish T 2013 A dual read-out assay to evaluate the potency of compounds active against Mycobacterium tuberculosis. PLoS One 8:e60531. doi:10.1371/journal.pone.0060531. 23593234 PMC3617142

[31] Lambert RJ, Pearson J 2000 Susceptibility testing: accurate and reproducible minimum inhibitory concentration (MIC) and non-inhibitory concentration (NIC) values. J Appl Microbiol 88:784–790. doi:10.1046/j.1365-2672.2000.01017.x. 10792538

[32] Chandrasekera NS, Berube BJ, Shetye G, Chettiar S, O’Malley T, Manning A, Flint L, Awasthi D, Ioerger TR, Sacchettini J, Masquelin T, Hipskind PA, Odingo J, Parish T 16 10 2017 Improved phenoxyalkylbenzimidazoles with activity against *Mycobacterium tuberculosis* appear to target QcrB. ACS Infect Dis. doi:10.1021/acsinfecdis.7b00112. PMC5727484 29035551

